# Trajectories of adherence to intravenous biological treatment in patients with inflammatory bowel disease: a longitudinal analysis

**DOI:** 10.3389/fphar.2024.1431035

**Published:** 2024-11-28

**Authors:** Xiuli Dong, Suyan Zhu, Yiyi Jin, Chaoqun Ren, Chunyan Chen

**Affiliations:** ^1^ Department of Gastroenterology and Hepatology, The First Affiliated Hospital of Wenzhou Medical University, Wenzhou, Zhejiang Province, China; ^2^ Departments of Pharmacy, The First Affiliated Hospital of Ningbo University, Ningbo, Zhejiang Province, China

**Keywords:** inflammatory bowel disease, adherence, biologics, intravenous, trajectories

## Abstract

**Background:**

Long-term biological therapies for inflammatory bowel disease (IBD) include infliximab and vedolizumab, which are administered intravenously. Although highly effective, non-adherence to these biologics is common and is associated with adverse sequelae and loss of response.

**Objective:**

In this study, we aim to characterize long-term intravenous biologic adherence trajectories among IBD patients and identify the factors linked with these trajectories.

**Methods:**

We conducted a retrospective multicenter study of IBD patients over 2 years to assess their adherence to infliximab and vedolizumab. The date of infusion was determined based on medical and pharmacy records. Using group-based trajectory modeling (GBTM), adherence trajectories were identified based on patients’ 90-day coverage of days over time. The effect of relevant variables on adherence behavior was assessed using multinomial regression analysis.

**Results:**

374 patients with IBD were included in the study, 68.2% males with a median age of 34.3 (IQR 28.0–44.4) years old. Three distinct adherence trajectories were identified for intravenous biologics: “consistent adherence” (n = 136, 36.4%), “slow decline” (n = 137, 36.6%) and “rapid decline” (n = 101, 27.0%). Compared with consistent adherence, concomitant use of aminosalicylates (OR 3.49, 95% CI 1.34–9.05) was associated with a significantly greater risk of rapid decline. Conversely, being married at the initiation of biologics (OR 0.43, 95% CI 0.19–0.95) and having been hospitalized within preceding years (OR 0.44, 95% CI 0.23–0.88) appeared to have a protective effect against rapid decline. Additionally, being male (OR 0.57, 95% CI 0.32–1.01) was found to be protective against slow decline.

**Conclusion:**

Distinct adherence patterns for infusion biologics among IBD patients have been identified, offering valuable insights to refine the design and timing of adherence interventions. However, only limited factors were found to be associated with specific adherence trajectories, revealing the complex nature of adherence behavior.

## Introduction

IBD, which encompasses crohn’s disease (CD) and ulcerative colitis (UC), is a chronic inflammatory condition characterized by alternating periods of remission and relapse (Loftus, 2004). Conventional therapy fails to control IBD in some patients, requiring biologic treatment instead (Rutgeerts et al., 2009). Due to the increasing number of biologics available for IBD therapy, clinicians may individualize treatment based on their own judgment, taking into account multiple factors, including adherence.

Infliximab and vedolizumab are widely accessible and utilized for the treatment of IBD. After an initial 6-week regimen of three doses, both are administered intravenously at 8-week intervals. The efficacious treatment targets achieved by infliximab and vedolizumab include endoscopic and clinical remission in IBD (Peyrin-Biroulet et al., 2015). To achieve these goals, patients must strictly adhere to their prescribed drug regimens, which is crucial for maintaining therapeutic efficacy of intravenous biologics. Nevertheless, the adherence to biologics is often poor and varies considerably depending on the study design, monitoring duration, and the definition of adherence (Haar et al., 2021; Martelli et al., 2017; Moran et al., 2019).

Medication adherence is frequently overestimated and oversimplified by traditional definitions, such as taking 80% of prescribed medications. Adherence encompasses multiple phases: timely initiation, correct implementation, and continuation throughout the intended duration of the prescription (Vrijens et al., 2012). In other words, non-adherence can manifest in diverse forms and follow varying trajectories, whereas traditional definitions like the proportion of days covered (PDC) may be insensitive to this dynamic nature.

Advanced statistical methods, such as group-based trajectory modeling (GBTM), have recently been employed to identify distinct longitudinal adherence trajectories (Alhazami et al., 2020). GBTM has proven to be a robust methodology that surpasses PDC in discriminating between different dynamic adherence experiences over time (Lo-Ciganic et al., 2016; Winn and Dusetzina, 2016). Furthermore, tailored interventions may be feasible based on the specific underlying causes of these trajectories.

To our knowledge, the adherence trajectories of biologic therapies in patients with IBD have not been studied.

By understanding risk factors for biologic nonadherence, individual treatment regimens can be developed, and patient outcomes can be improved. The primary objective of this study was to evaluate the trajectories of adherence to biologics among patients with IBD. As a secondary objective, we aimed to identify risk factors associated with biologics adherence trajectories.

## Methods

### Study design and data sources

We conducted a retrospective cohort study of patients who were diagnosed with IBD and initiated therapy with infliximab or vedolizumab from January 2010 to February 2022. We followed patients for 2 years after the “index date” to assess their adherence trajectories to biologics. In this context, the index date refers to the date when the first prescription for biologics was filled (Supplementary Figure S1). This study encompassed patients from two different sites: The First Affiliated Hospital of Ningbo University and The First Affiliated Hospital of Wenzhou Medical University. We excluded subjects with a follow-up period shorter than 2 years or those with fewer than three non-missing adherence values.

We obtained data from hospital medical records, pharmacy dispensing records, and outpatient clinic notes. When possible, information was cross-checked from multiple sources to minimize information bias. In addition to comprehensive demographics and clinical data, these data also included medical utilization information and medication details.

### Calculation adherence

**FIGURE 1:**
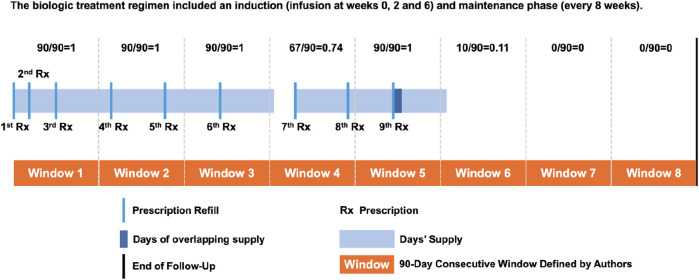
Visual representation of PDC calculation.

Proportion of days covered (PDC) was used as the primary measure of adherence in this study (Canfield et al., 2019). For each patient, 90-day consecutive windows were created from the index date to measure changes in adherence over time (Figure 1). Following that, each 90-day window’s PDC was calculated (Salmasi et al., 2021). In the same window, covered days of overlapping supplies were eliminated. For patients who permanently discontinued their medication, a PDC of zero was assigned for all subsequent time windows following the exhaustion of their last medication supply.

### Ananalysis plan

Categorical data were described by frequency and percentage, and continuous data were described by medians with interquartile ranges. Biologic adherence trajectories were identified using GBTM (Hickson et al., 2020; Nagin et al., 2024; Nagin and Odgers, 2010). This method identifies latent groups of individuals following a similar longitudinal trajectory, allowing evaluation of their frequency and associations with risk factors. As described below, GBTM assumes *a priori* the existence of at least two distinct trajectory groups, and the selection of more complex models is driven by both content expertise and statistical criteria (Nagin and Odgers, 2010).

To begin, we modeled adherence trajectories utilizing 2 to 5 groups with cubic polynomials. Models were sorted from best (largest Bayesian information criterion, i.e., closest to 0) to worst. The final trajectory model should meet the following criteria: (a) ≥10% of population assigned to each group; (b) an average posterior probability (APP) > 0.70 for each group; (c) the odds of correct classification (OCC) > 5 for each group; (d) upon visual inspection, narrow confidence intervals for estimated probability (Hernandez et al., 2019; Hickson et al., 2020; Nagin and Odgers, 2010).

The clinical and demographic factors of patients were summarized, and a multinomial logit model was constructed to identify factors associated with specific trajectory groups, as compared to the most adherent trajectory group. Data from all participants were incorporated into these models. Our analyses were conducted using IBM SPSS Statistics, Version 24.0 (IBM Corp, Armonk, NY) and R, version 4.1.2.

## Results

The study cohort consisted of 374 patients prescribed vedolizumab or infliximab with 2 years of follow-up, 240 (39.1%) patients were excluded due to having less than 3 adherence values or lost to follow-up (Supplementary Figure S2). In Table 1, the main characteristics of the study sample are summarized. Males accounted for the majority of patients (68.2%), with a median age of 34.3 [IQR 28.0–44.4] years. The median age at initiation of a biologic was 29.8 [IQR 23.7–40.6] years. Among infliximab-treated patients, 94.1% had CD. In contrast, among vedolizumab-treated patients, only 29.4% had CD. Disease duration at biologic initiation was 17.9 [IQR 2.9–58.8] months, with vedolizumab-treated patients exhibiting a longer disease duration compared to those treated with infliximab (vedolizumab: 43.7 [17.3–77.6] months, infliximab: 11.7 [1.2–42] months, P < 0.001). Approximately half of the patients received combination therapy, with a nearly equal proportion on immunomodulators (18.2%) or aminosalicylates (25.1%).

**TABLE 1:** Baseline characteristics of cohort.

Variable	Total (n = 374)	Infliximab (n = 306)	Vedolizumab (n = 68)
Male sex; yes (%)	255 (68.2)	214 (69.9)	41 (60.3)
Median age, years	34.3 (28.0–44.4)	32.8 (27.1–39.9)	45.4 (34.9–57.8)
Age at disease onset (years)	27.0 (21.0–36.4)	25.7 (20–32.1)	39.2 (28.4–51.1)
Age at biologics initiation (years)	29.8 (23.7–40.6)	27.7 (22.5–36)	42.8 (32.4–55.5)
Median duration of IBD since diagnosis, years (IQR)	6.4 (4.1–8.8)	6.4 (4–8.6)	5.4 (4–8.9)
Disease duration at biologic initiation, months (IQR)	17.9 (2.9–58.8)	11.7 (1.2–42)	43.7 (17.3–77.6)
Haemoglobin at biologics initiation (g/L)	127 (112–140)	126 (112–139)	127 (110.3–140.5)
Erythrocyte sedimentation rate (mm/h)	21 (10–36)	22 (11–37)	13.5 (6–28.5)
C-reactive protein at biologics initiation (mg/L)	6.8 (2.1–22.9)	9.1 (3–28.9)	3.2 (1.1–13.8)
Albumin at biologics initiation (g/L)	39.6 (35.2–43.6)	39 (35.1–43.0)	40 (34.6–44.2)
Body mass index at biologics initiation	19.3 (17.6–21.9)	19 (17.3–21.5)	20.8 (18.7–23.4)
Smoking status at biologics initiation; yes (%)	29 (7.8)	25 (8.2)	4 (5.9)
Married at biologics initiation; yes (%)	105 (28.1)	82 (26.8)	23 (33.8)
Past IBD-related surgery; yes (%)	133 (35.6)	126 (41.2)	7 (10.3)
Crohn’s Disease; yes (%)	308 (82.4)	288 (94.1)	20 (29.4)
Baseline nonbiologic IBD medications; yes (%)			
Aminosalicylates	157 (42.0)	114 (37.3)	43 (63.2)
Corticosteroids	77 (20.6)	56 (18.3)	21 (30.9)
Immunomodulators	71 (19.0)	62 (20.3)	9 (13.2)
Concomitant nonbiologic IBD medications; yes (%)			
Aminosalicylates	68 (18.2)	34 (11.1)	34 (50)
Corticosteroids	33 (8.8)	22 (7.2)	11 (16.2)
Immunomodulators	94 (25.1)	88 (28.8)	6 (8.8)
Disease location Crohn’s disease; yes (%)			
L1	89 (23.8)	78 (25.5)	11 (16.2)
L2	49 (13.1)	46 (15)	3 (4.4)
L3	153 (40.9)	149 (48.7)	4 (5.9)
L4	21 (5.6)	20 (6.5)	1 (1.5)
Disease behaviour Crohn’s disease; yes (%)			
B1	212 (56.7)	179 (58.5)	33 (48.5)
B2	102 (27.3)	90 (29.4)	12 (17.6)
B3	33 (8.8)	29 (9.5)	4 (5.9)
Perianal disease	167 (44.7)	157 (51.3)	10 (14.7)
Disease location ulcerative colitis; yes (%)			
E1	9 (2.4)	0 (0)	9 (13.2)
E2	19 (5.1)	9 (2.9)	10 (14.7)
E3	38 (10.2)	9 (2.9)	29 (42.6)

Disease location was assessed according to the Montreal classification at time of biologic initiation. Crohn’s disease: L1, ileal; L2, colonic; L3, ileocolonic; L4, upper gastrointestinal involvement; B1, non-stricturing, non-penetrating; B2, structuring; B3, penetrating. Ulcerative colitis: E1, proctitis; E2, left-sided colitis; E3, pancolitis; IQR: interquartile range.

**FIGURE 2:**
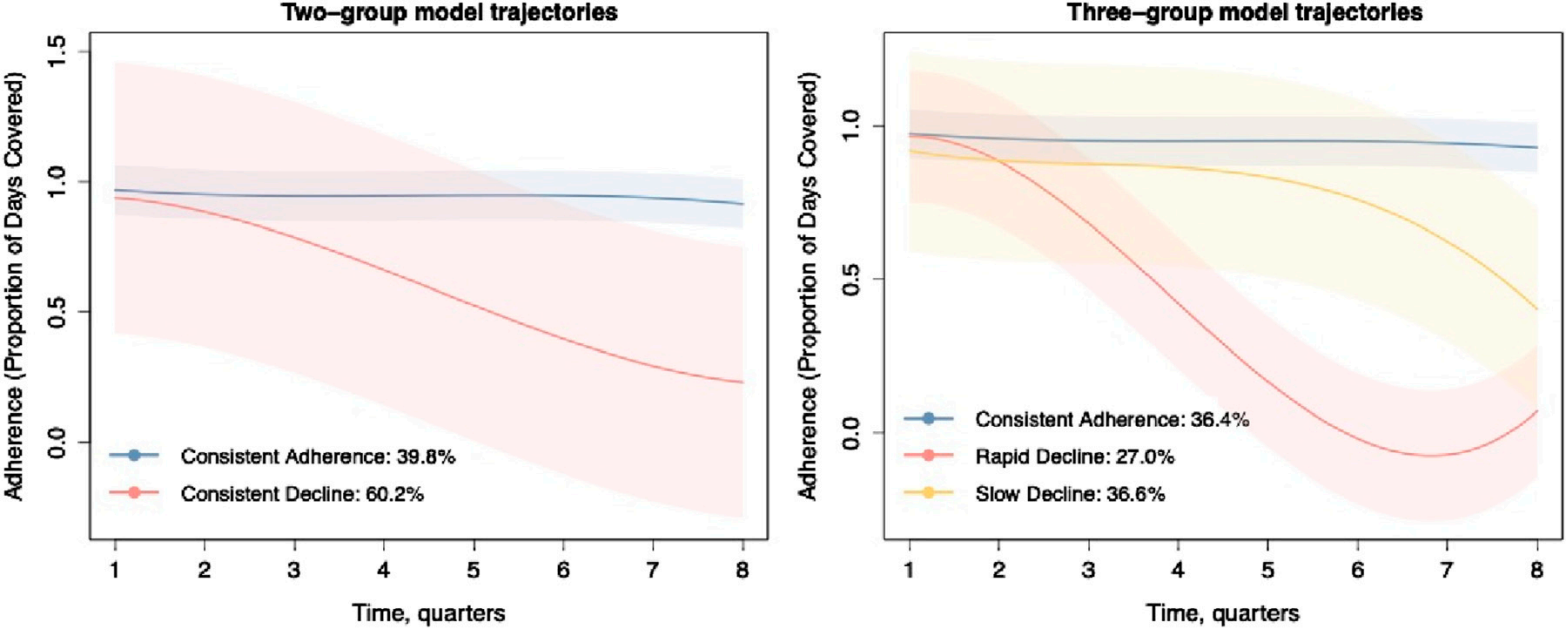
Adherence behavior to biologics with 2 and 3 groups over 2 years of observation.

Supplementary Table S1 demonstrates that the two-group solution yielded the largest Bayesian information criterion (BIC) value. However, the 2-class model produced wide confidence intervals for the estimated probabilities, indicating high uncertainty in the estimates and thus limited clinical significance (Figure 2). Based on the criteria mentioned in METHODS, we simultaneously identified a three-group trajectory model. This three-group model met all of Nagin’s criteria (OCC was >5, APP was >70%, and each group had >10% of our sample, Supplementary Table S1), balancing model complexity and goodness of fit to the sample data. Supplementary Figure S3 illustrates various biologics adherence trajectory models, ranging from 2 to 5 groups. Supplementary Figure S4 presents a spaghetti plot, a graphical representation of individual trajectories, for each of the groups in the final 3-group trajectory model, enabling visualization of the heterogeneity within and between groups. Supplementary Figure S5 shows the visual comparison of observed PDC versus the model predicted PDC for each of the 3 trajectories. The close proximation of the observed and predicted measures indicate good model fit.

According to their shape, we labeled the trajectories of the two-group model as “consistent adherence” (n = 149, 39.8%) and “consistent decline” (n = 225, 60.2%). Additionally, three-group model trajectories were labeled as “consistent adherence” (n = 136, 36.4%), “slow decline” (n = 137, 36.6%), and “rapid decline” (n = 101, 27.0%) (Figure 2). Consistently adherent patients demonstrated relatively high PDC scores, surpassing the threshold of 0.8 in both models over time. In the continuous decline trajectory of the two-group model, we observed that 60.2% of participants exhibited non-adherence to biologics, with adherence rates falling from 92% to 18%, indicating a high degree of variability in adherence behavior. To enhance clinical relevance, we introduced a new group to gain deeper insights into non-adherence patterns. In the three-group model trajectories, non-adherent trajectories were categorized as slow decline and rapid decline, accounting for 63.6% of the study population. This proportion is nearly identical to the continuous decline (60.2%) observed in the two-group model. For individuals experiencing rapid decline, their PDC plummeted to nearly zero within the first year, followed by late partial improvement that never reached an adherent level. In contrast, adherence progressively declined over time in the slow decline trajectory.

In addition, we have also conducted a sensitivity analysis, excluding those who were on concomitant immunomodulator therapy, to ensure that the outcomes measured were reflective of the biologic agent alone. A new set of trajectories has been identified in Supplementary Figure S6. The three new trajectories show a trend that is not very different from that of the main analysis.

**FIGURE 3:**
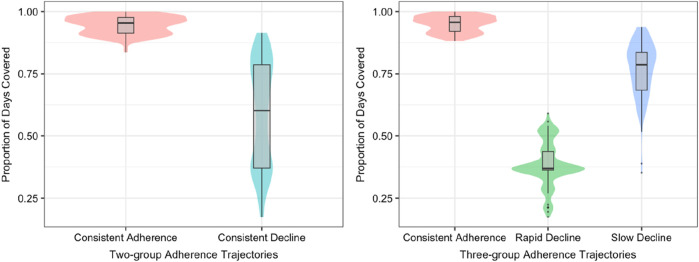
Mean observed adherence for patients assigned to each trajectory group.

As shown in Figure 3, each trajectory group’s mean PDC over time is distributed. The mean PDC observed was 0.96 ± 0.04 for consistent adherence and 0.59 ± 0.21 for consistent decline in two-group adherence model. In the three-group model trajectories, mean observed PDC was 0.95 ± 0.03 for consistent adherence, 0.77 ± 0.11 for slow decline, and 0.39 ± 0.09 for rapid decline. Patients with a consistent adherence trajectory exhibit a high mean PDC, which further validates the group assignments. Furthermore, to gain deeper insights into the factors contributing to non-adherence in GBTM-classified groups, we analyzed patients in each trajectory of the 3-group model who had an average PDC below 80%, and the results are presented in Supplementary Figure S7. A significantly higher rate of non-responsiveness to biologics was seen in patients with slow (17.6%) and rapid (25%) decline, compared to those adhering consistently (10%). Additionally, side effects were more common in these decline groups, further contributing to lower adherence (Supplementary Table S2).

**FIGURE 4:**
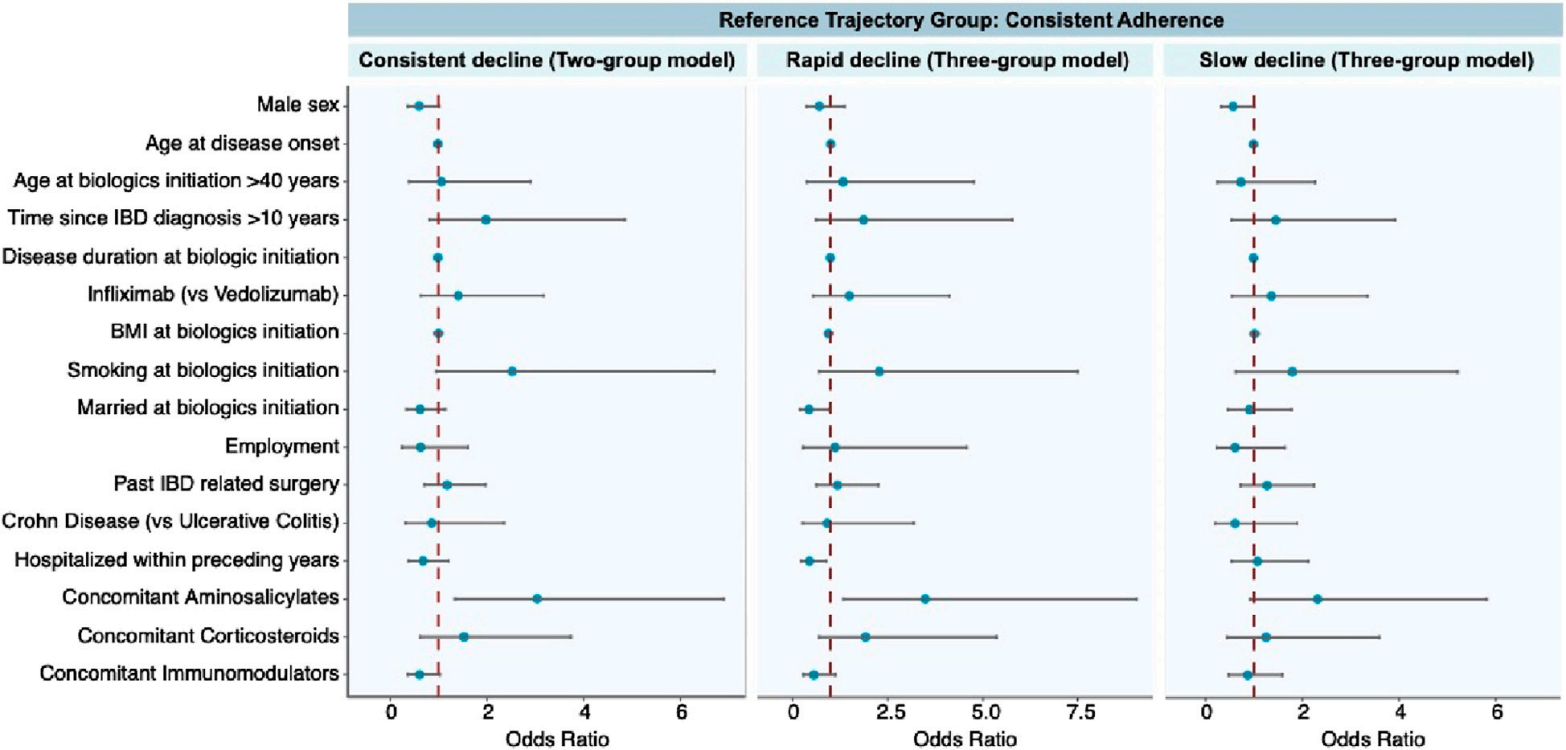
Factors associated with each distinct adherence trajectory.

The Supplementary Table S3 provides an overview of each trajectory’s key demographic and clinical characteristics. The results of multinomial logistic regression are presented in Figure 4, with consistent adherence as the reference group. A detailed description of the multinomial logit model can be found in Supplementary Table S4. Following are the factors significantly linked with each trajectory.

Consistent decline trajectory. The concomitant use of aminosalicylates was the only factor that was associated with a higher probability of consistent decline in the two-group model.

Rapid decline trajectory. Concomitant use of aminosalicylates was associated with a greater risk of rapid decline in the three-group model. Being married at the initiation of biologics therapy and having been hospitalized within preceding years appeared to have a protective effect against rapid decline.

Slow decline trajectory. Males were protective against slow decline in the three-group model. Biological types and disease classifications did not relate to any adherence patterns.

## Discussion

This study identified three adherence trajectories to biologics in patients with IBD: consistent adherence, slow decline, and rapid decline. The latter two trajectories, identified as non-adherence, differed in terms of the rate and timing of medication decline. This retrospective study offers valuable insights into the intricate issue of non-adherence among IBD patients, which can inform the design of interventions aimed at enhancing adherence. As far as we know, no trajectory models have been used to investigate the implementation phase of adherence to biologics in patients with IBD. Greenley et al. (2015) also attempted to apply a trajectory model to IBD patients, but their focus was primarily on oral thiopurine adherence. Our analysis has, for the first time, identified three adherence trajectories for infusion-based biologics over a span of 2 years.

The available IBD studies, which did not consider longitudinal adherence behaviors, demonstrated a wide range of adherence rates, varying from 30% to 90% (Haar et al., 2021; Li et al., 2023; Long et al., 2022; Lopez et al., 2013). In our study, non-adherence to biologics was observed in 60.2% of participants in the two-group model. To enhance clinical significance, we introduced an additional group to gain a more comprehensive understanding of non-adherence. In the three-group model trajectories, non-adherent trajectories were defined as slow decline and rapid decline, which accounted for 63.6% of the study population. A majority of IBD patients in our study do not adhere to biologics over time, indicating both disease activity and therapy response. Indeed, since biologics treatment is intended to achieve clinical and endoscopic remission, tapering or discontinuing these drugs should be determined by clinical needs and safety concerns. Specifically, for IBD treatment, the minimum level of adherence necessary to control symptoms is currently unknown, as it is for many other chronic diseases. Treatments for adult hypertension require minimum adherence rates of 80% (Haynes et al., 1976), while HIV treatment necessitates adherence rates of 95% (Liu et al., 2001). Further study is necessary to investigate in detail how drug-utilization behavior impacts effectiveness and safety.

We employed a multinomial logit model to examine the influence of various variables on the manifestation of each adherence pattern. While there were some exceptions, male gender generally correlated with lower non-adherence patterns, especially in cases of slow declines. Consistent with our findings, Kane et al. found that female gender appears to be a risk factor associated with non-adherence (Kane and Dixon, 2006). It is interesting to note that female gender is associated with non-adherence. However, based on other adherence literature, the male gender has been linked to a higher rate of non-adherence (Sewitch et al., 2003). There is no evidence to suggest that infliximab is less effective or less well tolerated in women, which could explain this discrepancy. Nevertheless, a recent systematic review revealed that women with IBD report poorer psychological wellbeing and less resilience than men. Additionally, they tend to develop more escape and avoidance strategies to cope with the disease (Truta, 2021).

We observed that concomitant use of aminosalicylates was correlated with a higher likelihood of rapid decline. Previous studies have also indicated that non-adherence to therapy may stem from polypharmacy, concerns regarding potential side effects, and the complex administration of topical aminosalicylates (such as difficulty in swallowing tablets or using enemas), all of which can undermine medication beliefs (Li et al., 2023; Magalhaes et al., 2014). Furthermore, a meta-analysis has demonstrated that patients with lower belief in the necessity of medication and higher concern about its potential harm are more likely to exhibit poor adherence (Adem et al., 2021). Variables that were protective against rapid decline in our population included being married at the initiation of biologics therapy and having been hospitalized within preceding years. Compared to unmarried patients, married patients demonstrated higher adherence. This may be attributed to a potentially elevated socioeconomic status among married patients, as well as a reduced emotional burden (Zhao et al., 2019). They may also receive support from their spouses or children, both emotionally and financially (Feng et al., 2020). Being hospitalized within preceding years demonstrated a protective effect against rapid decline. This finding has been previously documented in studies on adherence and may be attributed to an increased awareness of the consequences of non-adherence (Salmasi et al., 2021).

Overall, only a small number of variables were linked to a particular adherence trajectory. Further research, especially qualitative studies, is necessary to identify the psychosocial factors associated with different adherence patterns. To date, few effective strategies have been developed for improving biologic adherence among IBD patients (Abdullah et al., 2021; Haar et al., 2021). The results of this study can be used to tailor the content of adherence interventions for various forms of non-adherence. First, efforts to identify patients’ adherence trajectory early in therapy appear to be essential. Additionally, women were more likely to belong to the slow non-adherent trajectory, indicating the need for customized and targeted interventions specifically for women. A strong predictor of rapid non-adherence was the concomitant use of aminosalicylates. In light of this perceived risk, healthcare professionals can utilize this information to identify individuals at risk of non-adherence, who may then benefit from preventative interventions aimed at reducing their risk of non-adherence over time.

Limitations of the study should also be discussed. First, the retrospective nature of hospital-based data collection hindered our ability to identify other potential cofactors of medication adherence, including psychosocial factors, patient attitudes, and medication beliefs (Abdullah et al., 2021; Noor et al., 2023; Osterberg and Blaschke, 2005). Second, we were limited to utilizing only hospital-based health system variables, which meant that factors related to insurance coverage and income disparities could not be evaluated. Third, the current sample size was modest and may have limited our ability to detect additional latent classes and influencing factors. However, this seems somewhat unlikely, given that our sample size is comparable to those of other studies investigating latent trajectory groups within IBD populations (Greenley et al., 2015; Hommel et al., 2017). Nonetheless, further research employing a larger sample would be invaluable for replicating and extending the current findings. Lastly, given that demographic and clinical characteristics offered limited predictive utility in determining adherence trajectory class, future research would benefit from exploring additional domains that may influence adherence trajectory patterns.

## Conclusion

This retrospective study identified three distinct adherence trajectories among IBD patients, based on the rate and timing of medication decline. These trajectories included consistent adherence, slow decline, and rapid decline. According to these findings, adherence can be improved by tailoring interventions to different patterns of non-adherence, rather than taking a generic approach. In general, we found only a few variables that were uniquely associated with a particular adherence trajectory. Further studies are warranted to investigate the relationship between adherence behavior and effectiveness and safety outcomes.

## Data Availability

The raw data supporting the conclusions of this article will be made available by the authors, without undue reservation.
