# Supplementary material for: Trajectories of adherence to intravenous biological treatment in patients with inflammatory bowel disease: a longitudinal analysis
Source: Front Pharmacol. 2024 Nov 28;15:1431035. doi: 10.3389/fphar.2024.1431035 (PMC11634588; doi:10.3389/fphar.2024.1431035)
Supplement: Supplementary file 1 [file Table1.DOCX]

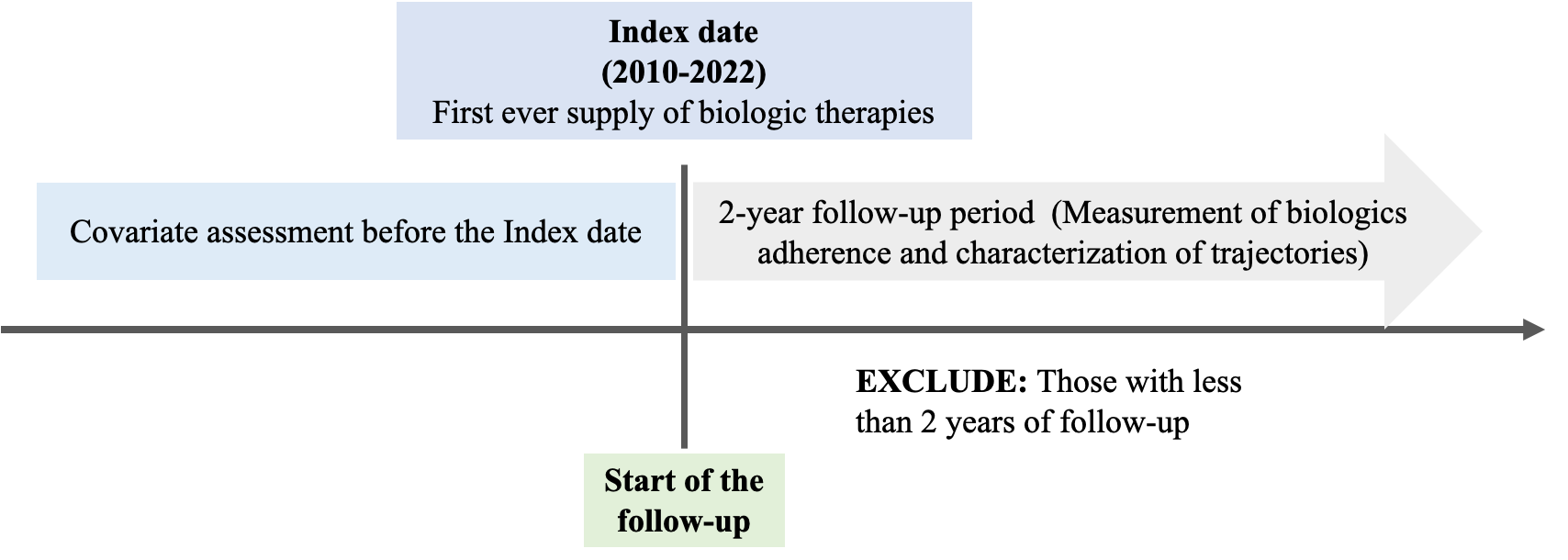


Figure S1. Overview of the study design


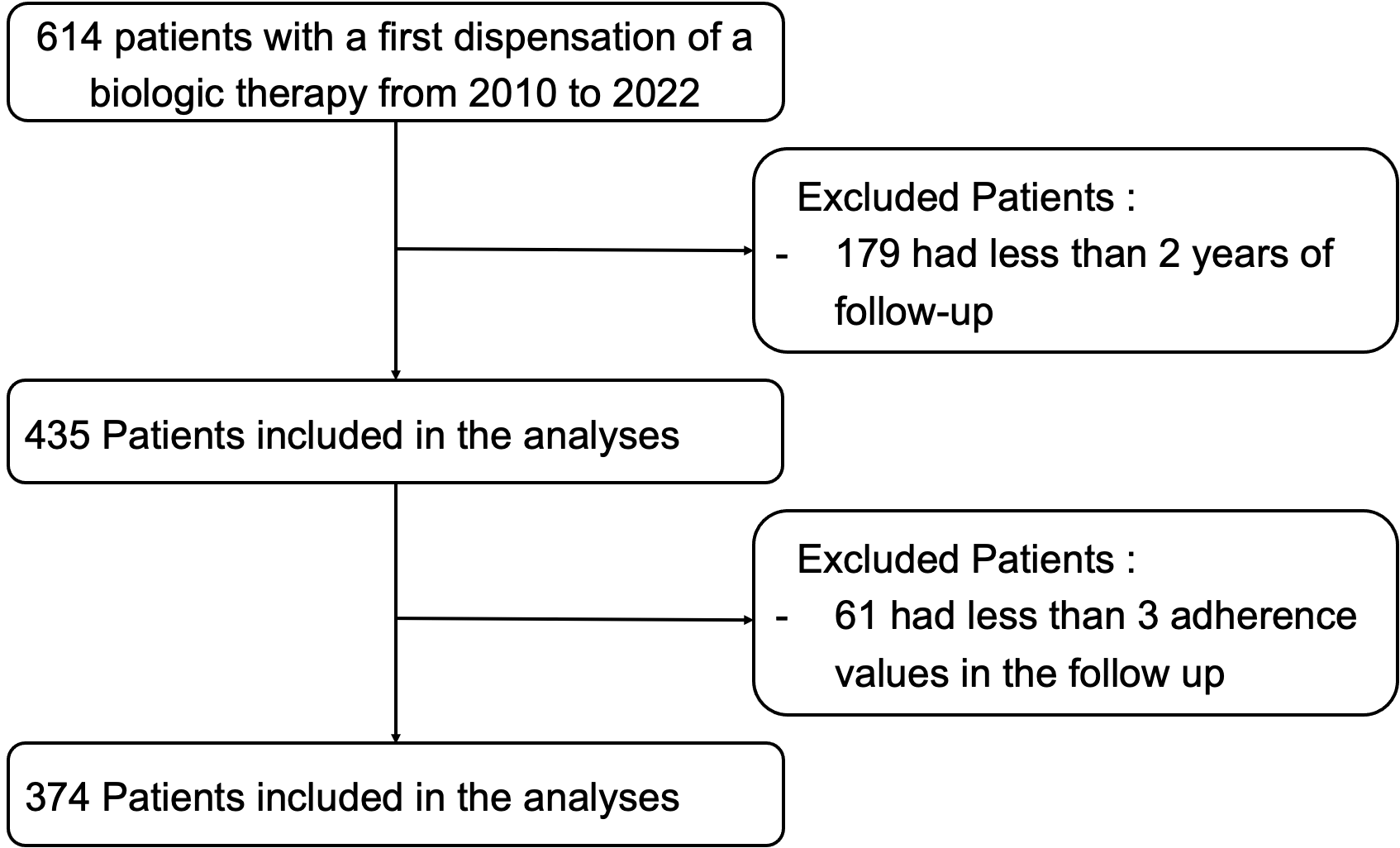


Figure S2. Study flow chart

Table S1: Characteristics of the selected model.

| model | APP | | | | | AIC | BIC | Min OCC |
| --- | --- | --- | --- | --- | --- | --- | --- | --- |
|  | G1 | G2 | G3 | G4 | G5 |  |  |  |
| traj_2 | 0.99 | 1.00 | NA | NA | NA | -976.41 | -910.36 | 199.06 |
| traj_3 | 0.99 | 0.99 | 0.98 | NA | NA | -1925.68 | -1823.62 | 89.72 |
| traj_4 | 0.99 | 0.98 | 0.93 | 0.96 | NA | -2102.03 | -1969.95 | 81.35 |
| traj_5 | 0.99 | 0.99 | 0.93 | 0.93 | 0.96 | -2102.03 | -1969.95 | 96.26 |

APP: average posterior probability; BIC: Bayesian information criterion; AIC: Akaike information criterion; OCC: odds of correct classification. NA: not applicable.

Figure S3. Biologics adherence trajectory models with 2 to 5 groups

Figure S4. Spaghetti plots of individual adherence patterns for each adherence group in the final 3-group trajectory model. Individual and mean trajectories are displayed in grey and black, respectively.

Figure S5. Goodness of fit of the final 3-group trajectory model

Figure S6. Sensitivity analysis of the 3-group trajectory model

Figure S7. Reasons for the decrease in adherence in the 3-group trajectory model.

Table S2: The Reasons and proportions for the decrease in adherence in the 3-group trajectory model.

|  | Consistent adherence | Rapid decline | Slow decline |
| --- | --- | --- | --- |
| Non-responsive to biologics; yes (%) | 6 (10.0) | 11 (25.0) | 12 (17.6) |
| Other or unknown; yes (%) | 1 (1.7) | 2 (4.5) | 5 (7.4) |
| Pregnancy; yes (%) | 1 (1.7) | 0 | 1 (1.5) |
| Remission/Response; yes (%) | 49 (81.7) | 17 (38.6) | 36 (52.9) |
| Self-discontinuation of medication; yes (%) | 0 | 3 (6.8) | 2 (2.9) |
| Side effect; yes (%) | 0 | 9 (20.5) | 4 (5.9) |
| Switching to other biologics; yes (%) | 3 (5.0) | 1 (2.3) | 6 (8.8) |
| Switching to other oral medications; yes (%) | 0 | 1 (2.3) | 2 (2.9) |

Table S3: Characteristics of patients in each group.

|  | Two-group trajectory model | | Three-group trajectory model | | |
| --- | --- | --- | --- | --- | --- |
|  | Consistent adherence | Consistent  Decline | Consistent adherence | Rapid  Decline | Slow  Decline |
| Male sex | 107 (71.8) | 148 (65.8) | 98 (72.1) | 68 (67.3) | 89 (65.0) |
| Age at disease onset (years) | 26.9 (21.2-34.5) | 27.0 (20.7-36.8) | 27.3 (21.0-36.0) | 27.9 (21.9-40.0) | 25.0(20.0-34.0) |
| Age at biologics initiation >40 (years) | 37 (24.8) | 61 (27.1) | 36 (26.5) | 32 (31.7) | 36 (26.3) |
| Time since IBD diagnosis >10 (years) | 28 (18.8) | 44 (19.6) | 27 (19.9) | 19 (18.8) | 26 (19.0) |
| Disease duration at biologic initiation, months | 23.4 (6.3-65.2) | 16.1 (0.8-57.9) | 24.4 (7.2-66.4) | 11.0 (0.5-56.0) | 16.4 (1.8-58.8) |
| Infliximab (vs vedolizumab) | 123 (82.6) | 183 (81.3) | 112 (82.4) | 80 (79.2) | 114 (83.2) |
| Body mass index at biologics initiation | 19.6 (17.6-21.7) | 19.1 (17.5-22) | 19.7 (17.7-21.9) | 19 (17.3-21.3) | 19.5 (17.7-22.0) |
| Smoking status at biologics initiation; yes (%) | 9 (6.0) | 20 (8.9) | 9 (6.6) | 9 (8.9) | 11 (8.0) |
| Married at biologics initiation; yes (%) | 48 (32.2) | 57 (25.3) | 45 (33.1) | 20 (19.8) | 40 (29.2) |
| Employment; yes (%) | 131 (87.9) | 185 (82.2) | 119 (87.5) | 89 (88.1) | 111 (81.0) |
| Past IBD‐related surgery; yes (%) | 53 (35.6) | 80 (35.6) | 48 (35.3) | 33 (32.7) | 52 (38.0) |
| Crohn’s Disease; yes (%) | 130 (87.2) | 178 (79.1) | 119 (87.5) | 77 (76.2) | 112 (81.8) |
| Hospitalized within preceding years | 124 (83.2) | 170 (75.6) | 113 (83.1) | 67 (66.3) | 114 (83.2) |
| Concomitant Aminosalicylates | 14 (9.4) | 54 (24.0) | 13 (9.6) | 30 (29.7) | 25 (18.2) |
| Concomitant Corticosteroids | 8 (5.4) | 25 (11.1) | 7 (5.1) | 15 (14.9) | 11 (8.0) |
| Concomitant Immunomodulators | 43 (28.9) | 51 (22.7) | 37 (27.2) | 21 (20.8) | 36 (26.3) |

Table S4: Factors associated with exhibiting each adherence trajectory. Results of multinomial logistic regression

|  | Odds Ratio (95% Confidence interval)  Reference: Consistent adherence | | |
| --- | --- | --- | --- |
|  | Two-group model | Three-group model | |
|  | Consistent decline | Rapid decline | Slow decline |
| Male sex | 0.60 (0.36, 1.02) | 0.70 (0.36, 1.36) | 0.57 (0.32, 1.01) |
| Age at disease onset (years) | 0.99 (0.96, 1.03) | 1.00 (0.96, 1.05) | 0.99 (0.95, 1.03) |
| Age at biologics initiation >40 (years) | 1.06 (0.39, 2.90) | 1.33 (0.37, 4.76) | 0.73 (0.24, 2.26) |
| Time since IBD diagnosis >10 (years) | 1.98 (0.81, 4.85) | 1.87 (0.61, 5.77) | 1.45 (0.54, 3.91) |
| Disease duration at biologic initiation, months | 0.99 (0.98, 1.00) | 0.99 (0.98, 1.00) | 0.99 (0.99, 1.00) |
| Infliximab (vs vedolizumab) | 1.41 (0.63, 3.17) | 1.49 (0.54, 4.13) | 1.36 (0.55, 3.35) |
| Body mass index at biologics initiation | 1.00 (0.92, 1.08) | 0.94 (0.85, 1.04) | 1.01 (0.93, 1.10) |
| Smoking status at biologics initiation; yes (%) | 2.52 (0.95, 6.70) | 2.28 (0.69, 7.50) | 1.79 (0.62, 5.21) |
| Married at biologics initiation; yes (%) | 0.62 (0.34, 1.14) | 0.43 (0.19, 0.95) | 0.91 (0.46, 1.77) |
| Employment; yes (%) | 0.63 (0.25, 1.61) | 1.12 (0.27, 4.58) | 0.61 (0.23, 1.64) |
| Past IBD‐related surgery; yes (%) | 1.18 (0.71, 1.97) | 1.18 (0.62, 2.26) | 1.27 (0.72, 2.23) |
| Crohn’s Disease; yes (%) | 0.86 (0.32, 2.35) | 0.91 (0.26, 3.19) | 0.61 (0.20, 1.89) |
| Hospitalized within preceding years | 0.68 (0.38, 1.21) | 0.44 (0.23, 0.88) | 1.07 (0.54, 2.12) |
| Concomitant Aminosalicylates | 3.04 (1.34, 6.89) | 3.49 (1.34, 9.05) | 2.31 (0.92, 5.81) |
| Concomitant Corticosteroids | 1.53 (0.62, 3.74) | 1.92 (0.69, 5.36) | 1.25 (0.44, 3.59) |
| Concomitant Immunomodulators | 0.61 (0.36, 1.03) | 0.56 (0.28, 1.12) | 0.87 (0.48, 1.58) |
